# The role of microbiota in the development and treatment of gastric cancer

**DOI:** 10.3389/fonc.2023.1224669

**Published:** 2023-09-29

**Authors:** Yiwen Wang, Wenjie Han, Na Wang, Mengzhen Han, Meng Ban, Jianying Dai, Yuesheng Dong, Tao Sun, Junnan Xu

**Affiliations:** ^1^Department of Breast Medicine 1, Cancer Hospital of China Medical University, Liaoning Cancer Hospital, Shenyang, China; ^2^Department of Pharmacology, Cancer Hospital of China Medical University, Liaoning Cancer Hospital, Shenyang, China; ^3^Department of Bioinformatics, Kanghui Biotechnology Co., Ltd., Shenyang, China; ^4^School of Bioengineering, Dalian University of Technology, Dalian, Liaoning, China; ^5^Department of Oncology Medicine, Key Laboratory of Liaoning Breast Cancer Research, Shenyang, Liaoning, China

**Keywords:** gastric cancer, microbiota, Helicobacter pylori (HP), Epstein-Barr virus (EBV), immunotherapy

## Abstract

The stomach was once considered a sterile organ until the discovery of *Helicobacter pylori (HP)*. With the application of high-throughput sequencing technology and macrogenomics, researchers have identified fungi and fivemajor bacterial phyla within the stomachs of healthy individuals. These microbial communities exert regulatory influence over various physiological functions, including energy metabolism and immune responses. *HP* is a well-recognized risk factor for gastric cancer, significantly altering the stomach’s native microecology. Currently, numerous studies are centered on the mechanisms by which *HP* contributes to gastric cancer development, primarily involving the CagA oncoprotein. However, aside from exogenous infections such as *HP* and EBV, certain endogenous dysbiosis can also lead to gastric cancer through multiple mechanisms. Additionally, gut microbiota and its metabolites significantly impact the development of gastric cancer. The role of microbial therapies, including diet, phages, probiotics and fecal microbiota transplantation, in treating gastric cancer should not be underestimated. This review aims to study the mechanisms involved in the roles of exogenous pathogen infection and endogenous microbiota dysbiosis in the development of gastric cancer. Also, we describe the application of microbiota therapy in the treatment and prognosis of gastric cancer.

## Introduction

Gastric cancer is the fifth most common cancer and the third leading cause of cancer-related deaths worldwide (1). Gastric cancer rarely detects in its early stage, and the lack of effective biomarkers can accurately diagnose early gastric cancer and monitor prognosis during the adjuvant stage, which results in higher mortality rates, with only one in five patients surviving more than five years after being diagnosed with gastric cancer (2). Consequently, the development of more accurate and accessible biomarkers is necessary to aid in the early-stage diagnosis of gastric cancer and to monitor relapse.

The microbiota in the human body comprises viruses, fungi and bacteria. High-fat diets, antibiotics, exogenous microbial infections and host genetics can lead to dysbiosis (3–5). The definition of dysbiosis is a change in the composition and function of the microbiota (5). A healthy and stable microbiota inhibits cancer development, whereas a dysbiotic microbiota has a limited protective effect on the host and promotes cancer development. Gastric cancer is the result of the interaction between the dysbiosis of the gastric microbiota and the host gastric epithelial cells (6). Moreover, as gastric cancer progresses, damage to the gastric mucosal barrier and persistent chronic inflammation will further promote dysbiosis (7).

*Helicobacter pylori (HP)* infection is one of the riskiest factors for gastric cancer (8). The majority of intestinal-type gastric cancers are associated with *HP*. *HP* infection accelerates the progression from atrophic gastritis (AG) to intestinal metaplasia (IM) and eventually to gastric cancer. Furthermore, individuals with *HP* infection have a 3-6 fold higher risk of developing gastric cancer than those without *HP* infection (2, 9). In recent years, viruses such as Epstein-Barr virus (EBV), Hepatitis B virus (HBV), and John Cunningham virus (JCV) have also attracted attention for their role in gastric cancer carcinogenesis. In addition to exogenous pathogen infections, some dysbiotic endogenous microbiota such as *Lactobacillus*, *F. nucleatum*, and *Candida albicans* can also be involved in developing gastric cancer through various mechanisms. Metabolites from the gut microbiota influence gastric cancer progression and improve anticancer therapy for gastric cancer patients. Microbiota therapy will be an essential target for future cancer treatment. In this review, the direct and indirect mechanisms by which exogenous infections and endogenous dysbiosis contribute to gastric cancer development are examined first. Next, the effects of oral and gut microbiota on gastric cancer are explored. Finally, the application of microbiota therapy in treating gastric cancer, including diet, bacteriophages, probiotics, and fecal microbiota transplantation (FMT), is discussed. Our goal is to investigate the impact of microbiota on gastric cancer, which could provide new insights for early screening and subsequent treatment of gastric cancer.

## Gastric microbiota

### Composition of the gastric microbiota in healthy individuals

Due to its high acidity, the stomach was once regarded as a sterile organ and unsuitable for microbial colonization. However, the discovery of *HP* led to the realization that some microbes could indeed colonize the stomach (2). Advancements in high-throughput sequencing technologies in microbiology have facilitated the discovery of an increasing number of exogenous and endogenous microbiota. The normal human stomach harbors a diverse microbiota, with over 130 lineages representing 7 to 13 bacterial phyla, of which 5 are the most dominant, including Proteobacteria, Firmicutes, Bacteroidetes, Actinobacteria, and Fusobacteria (10, 11). Fungi, a common yet easily overlooked component of gastric microbiota, have also been identified. Investigations of the gastric environment in healthy individuals, patients with chronic gastritis (CG), and gastric ulcers have detected hundreds of fungal strains in the stomach. Fungi may possess an effective acid tolerance mechanism, enabling them to proliferate more efficiently in a gastric environment with a pH of only 1.4 compared to bacteria. Researchers have detected fungal species, such as *Candida tropicales* and *Candida lusitanae*, in addition to the most common *Candida albicans*. Fungi usually colonize areas like the gastric mucosa and stomach contents (12, 13).

Both bacteria and fungi in the human stomach play crucial roles in various physiological activities, including energy metabolism, nutrient absorption, immune response, and nervous system regulation (14). However, some studies have observed differences in the flora of the gastric mucosa and gastric juice (8, 10, 11, 15, 16). Due to swallowing in the oral cavity and reflux in the duodenum, the gastric juice may contain more bacteria than the gastric mucosa (17). Although most current gastric flora studies have sampled the gastric mucosa, bacteria in the gastric juice should not be overlooked. These bacteria can contribute to the proper functioning of the gastrointestinal system but may also cause some adverse reactions.

### Composition of the gastric microbiota in gastric cancer patients

The results are diverse for changes in the diversity and abundance of microbiota in gastric cancer. Some studies showed that the abundance and diversity of the gastric microbiota did not change gradually with the development of gastric cancer (18, 19). Other studies have taken the opposite view, suggesting that the abundance and diversity of the microbiota decrease as gastric cancer progresses, which is what most studies have concluded (20–22). Another study has shown a significant increase in the abundance and diversity of the microbiota in cancerous tissues compared to paracancerous tissues. They also found that oral bacteria (such as *Peptostreptococcus*, *Streptococcus*, and *Fusobacterium*) dominated the cancerous tissues, and lactic acid-producing bacteria (such as *Lactococcus lactis* and *Lactobacillus brevis*) were enriched in the paracancerous tissues (23). However, Liu et al. found that *Prevotella melanogenic*, *Streptococcus anginosus* and *Propionibacterium acnes* were enriched in cancerous tissues compared to normal and paracancerous tissues. In contrast, the abundance of *HP*, *Prevotella copri* and *Bacteroides uniformis* was significantly reduced in cancerous tissues (24).

Many current studies exist on the microbiota of gastric cancer patients and controls. However, their results varied widely, and some were even contradictory, possibly due to different sample types, study populations, sequencing technologies and analysis methods **(** **Table 1****)**. However, it is undeniable that there is a significant difference in the microbial composition of gastric cancer patients compared to controls. In general, the microbiota of gastric cancer patients shows a decreased abundance of *HP*, a significant enrichment of the oral bacteria, and reduced diversity and abundance of the microbiota.

**Table 1:** Studies on the difference in gastric microbiota composition between patients with gastric cancer and controls.

Authors/Year	Country	Sample type	Study group	Richness/Diversity	Main differences in microbiota
Peng, 2023 (22)	China	Gastric juice	22 HC (Gastritis), 22 GPL (AG, IM, and adenomatous polyp), and 16 GC	The richness and diversity of gastric microbiota decreased gradually from HC, GPL to GC.	• Phylum level: From HC, GPL to GC: Proteobacteria and Spirochaetota↓, Firmicutes↑ • Genus level: *Vulcaniibacterium* and *Sphingomonas* were common in HC. From HC, GPL to GC: *Treponema*, *Campylobacter*, and *Neisseria*↓, *Lactobacillus* and *Streptococcus*↑
He, 2022 (17)	China	Gastric mucosal biopsy and gastric juice	61 SG, 55 IM, and 64 GC	The diversity of GM was significantly lower than that of GF. As the disease progressed, the differences between GM and GF in GC were significantly reduced compared with SG and IM.	• Enriched phyla: GF: Bacteroidetes, Fusobacteria and Proteobacteria GM: Firmicutes • Enriched genera: GM: *HP*, *Lactobacillus*, *Lactococcus*, and *Bacillus* GF: *Neisseria*, *Haemophilus*, *Streptococcus*, and *Gemella* • Compared to SG and IM, *Lactobacillus* was significantly increased in GC.
Sun, 2022 (21)	China	Gastric mucosal biopsy and gastric juice	56 SG, 9 AG, 27 IM, 29 Dys, and 13 GC (All patients were *HP-*)	The richness and diversity of gastric microbiota decreased gradually from SG, AG, IM, Dys to GC.	• Phylum level: The abundance of Firmicutes: GC > IM and Dys The abundance of Bacteroidetes: Dys < SG The abundance of Proteobacteria: IM and Dys > other stages • Genus Level: The abundance of *Streptococcaceae* and *Lactobacillaceae*: GC > SG AG to Dys: *Burkholderaceae*↑, *Streptococcaceae* and *Prevotellaceae*↓ The dominant genus of IM and Dys: *Ralstonia* and *Rhodococcus*
Deng, 2021 (19)	China	Gastric mucosal biopsy	25 SG (All patients were *HP*-, antrum 10, corpus 7, and cardia 8) and 34 GC (9 *HP*+ and 25 *HP*-, antrum 19 and corpus 15)	There was no significant difference in the diversity and richness of gastric microbiota between SG and GC.	• Enriched order: Antrum: *HP*- GC: *Peudomonodales* and *Erysipelotrichales* *HP*+ GC: *Neisseriales* SG: *Actinomycetales*, *Enterobacteriales* and *Pasteurellales* Corpus: *HP*- GC: *Peudomonodales* *HP*+ GC: *Campylobacterales* and *Bacteroidales* SG: *Enterobacteriales*, *Actinomycetales*, and *Burkholderiales*
Zhang, 2021 (18)	China	Gastric mucosal biopsy	17 SG, 10 AG, 5 IN, and 15 GC	In different stages of GC, the richness and diversity of gastric microbiota did not change gradually, and the richness of AG was the highest.	• Enriched genera: SG to GC: Oral bacteria (*Slackia*, *Selenomonas*, *Bergeyella*, and *Capnocytophaga*) SG: *Anaerobacillus*, *Bacillus*, *Massilia*, and *Rhodobacter* AG: *Alloprevotella*, *Pelomonas*, *Ralstonia*, *Clocibacterium*, and *Deinococcus* IN: Intestinal commensal bacteria (*Romboutsia, Fusicatenibacter*, *Prevotellaceae-Ga6A1group*, and *Intestinimonas*) GC: Oral bacteria (*Parvimonas*, *Eikenella* and *Prevotella-2*) Environmental bacteria (*Kroppenstedtia*, *Lentibacillus*, and *Oceanobacillus*)
Wang, 2020 (20)	China	Gastric mucosal biopsy	30 HC, 21 CG, 27 IM, 25 IN, and 29 GC	The richness and diversity of gastric microbiota decreased gradually from HC, CG, IM, IN to GC.	• Enriched phyla: CG and IM: Acidobacteria, Gemmatimonadetes, Proteobacteria, and Verrucomicrobia IN and GC: Actinobacteria, Bacteriodes, Firmicutes, Fusobacteria, SR1, and TM7 • Enriched genera: HC: *Acinetobacter* and *Pseudomonas* CG and IM: *Halomonas*, *Shewanella*, *Aquincola* and *Sphingomonas* IN: *Granulicatella*, *Porphyromonas*, *unclassified Gemellaceae*, *Rothia*, and *Fusobacterium* GC: *HP* and *Lactobacillus* IN and GC: *Streptococcus*, *Prevotella*, and *Veillonella*
Chen, 2019 (23)	China	Gastric mucosal biopsy	62 pairs of matched cancer and para-cancerous tissues	The richness and diversity of microbiota in cancer tissues were significantly increased compared with that in para-cancerous tissues.	• Phylum level: Para-cancerous tissues: Firmicutes, Bacteroidetes, Actinobacteria, Acidobacteria and Fusobacteria↓, Proteobacteria↑ • Genus level: Cancer tissues: Oral bacteria (*Peptostreptococcus*, *Streptococcus*, and *Fusobacterium*)↑ Para-cancerous tissues: Lactic acid-producing bacteria (*Lactococcus lactoactis* and *Lactobacillus brevis*)↑
Liu, 2019 (24)	China	Gastric mucosal biopsy	276 GC (230 normal, 247 para-cancerous and 229 cancer tissues)	The richness and diversity of the microbiota showed a clear downward trend from normal to para-cancerous to cancer tissues.	•Enriched genera: Para-cancerous and cancer tissues: *B. fragilis* and *A. muciniphila* Para-cancerous tissues: *HP*, *Halomonas* and *Shewanella* Cancer tissues: *S. anginosus*, *Prevotella melaninogenica*, *Streptococcus anginosus*, *Selenomonas*, *Fusobacterium*, *Propionibacterium*, and *Corynebacterium* • Reduced genera: Cancer tissues: *Prevotella copri* and *Bacteroides* Normal and para-cancerous tissues: *S. anginosus*
Coker, 2018 (25)	China	Gastric mucosal biopsy	21 SG, 23 AG, 17 IM and 20 GC (biopsies of GC patients were from cancer and para-cancerous tissues)	Compared with other stages, the richness and diversity of the microbiota in GC decreased.	• The oral microbiota was significantly enriched in GC. The most important are *Peptostreptococcus stomatis*, *Slackia exigua*, *Parvimonas micra*, *Streptococcus anginosus*, and *Dialister pneumosintes*, which can be biomarkers to differentiate between GC and SG.
Ferreira, 2018 (26)	Portugal	Gastric mucosal biopsy	81 CG and 54 GC	GC showed reduced microbiota diversity.	• Phylum level: GC: Proteobacteria, Firmicutes and Actinobacteria↑ • Genus level: CG: *HP*, *Neisseria*, *Prevotella* and *Streptococcus*↑ GC: *HP*↓, *Achromobacter*, *Citrobacter*, *Phyllobacterium*, *Clostridium*, *Rhodococcus*, and *Lactobacillus*↑

SG, Superficial Gastritis; AG, Atrophic Gastritis; IM, Intestinal Metaplasia; GC, Gastric Cancer; CG, Chronic Gastritis; *HP*, *Helicobacter pylori*; *B. fragilis*, *Bacteroides fragilis*; *A. muciniphila*, *Akkermansia muciniphila*; HC, Healthy Controls, IN, Intraepithelial Neoplasia; *HP+*, *Helicobacter pylori positive*; *HP-*, *Helicobacter pylori negative*; Dys, Dysplasia; GPL, Gastric Precancerous Lesions; GM, Gastric mucosa; GF, Gastric fluid; ↑, increased; ↓, decreased.

### Effect of *HP*-positive and *HP*-negative gastric cancer on the gastric microbiota

*HP* is a gram-negative bacterium that parasitizes the human stomach. The colonization of *HP* can lead to dysbiosis. The diversity and abundance of the gastric microbiota decrease after infection with *HP* (27). *HP*-positive gastric cancers demonstrate an abundance of *Lactobacillus*, *Achromobacter*, *Citrobacter*, *Clostridium*, and *Rhodococcus* but lack *HP* and *Neisseria* compared to other gastric diseases (26). A recent meta-analysis suggests that eradicating *HP* can restore the diversity of the gastric microbiota and reduce the risk of gastric cancer (28). However, some studies contradict this result by suggesting that eradicating *HP* does not entirely prevent the development of gastric cancer, which indicates that other microorganisms in the stomach besides *HP* are also associated with gastric cancer carcinogenesis (29). Lertpiriyapong et al. demonstrated this hypothesis with INS-GAS mice. They found that *HP* mice colonized by complex or restricted microbiota were more susceptible to *HP*-induced gastric cancer than germ-free mice (30). This result suggests that after *HP* infection of the gastric epithelium causes dysbiosis, *HP* acts synergistically with the dysbiotic microbiota to promote gastric carcinogenesis.

**Figure 1:**
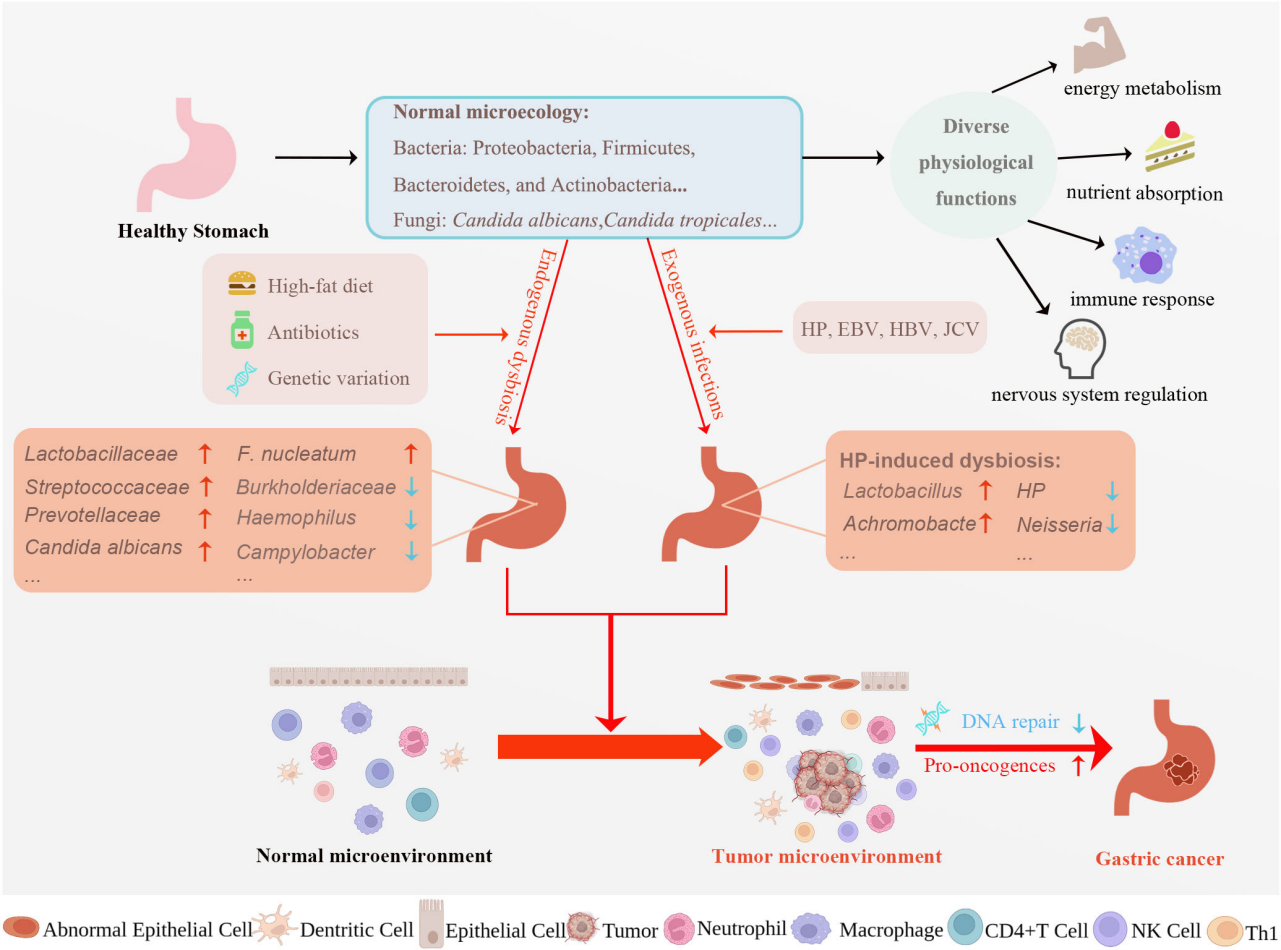
Factors affecting gastric microecological dysbiosis and potential mechanisms underlying microecological dysbiosis causing gastric carcinogenesis. A multitude of microorganisms resides in the stomach, creating normal gastric microecology. Normal microecology plays a vital role in various physiological activities such as energy metabolism, nutrient absorption, immune response, and nervous system regulation. High-fat diet, antibiotics, genetic variation, and exogenous infections can all dysregulate normal gastric microecology, turning the normal microenvironment into the tumor microenvironment and eventually leading to gastric cancer development. Exploring the mechanism of gastric cancer progression from the perspective of gastric microecology is essential for early diagnosis, treatment, and prognosis of gastric cancer. *HP, Helicobacter pylori*; EBV, Epstein-Barr virus; HBV, Hepatitis B virus; JCV, John Cunningham virus, *F. nucleatum, Fusobacterium nucleatum;* Th, helper T cell; CTL, Cytotoxic T lymphocyte. Created with BioRender.com. (accessed on 17 May 2023).

Although *HP* is recognized as a type 1 carcinogen by the World Health Organization (WHO), not all gastric cancers are caused by *HP* infection. *HP*-negative gastric cancers also account for a percentage ranging from 0.7% to 47.8% of gastric cancer patients (31). The role of microbiota in the development of *HP*-negative gastric cancer has attracted increasing attention. In *HP*-negative gastric cancer, Kim et al. found there was a significant increase in the abundance and diversity of *Lactobacillaceae*, *Streptococcaceae*, and *Prevotellaceae* and a decrease in the numbers of *Burkholderiaceae*, *Haemophilus*, and *Campylobacter* (32) **(** **Figure 1****)**. These findings are inconsistent in related studies, but the increased abundance of *Lactobacillaceae* was detected in almost all patients with gastric cancer. *Lactobacillaceae* is a nitrosating bacterium, and *Haemophilus* is a nitrate-reducing bacterium. An increase in the abundance of *Lactobacillaceae* and a decrease in the abundance of *Haemophilus* can increase the formation of N-nitroso compounds, which can cause persistent inflammation and promote the development of gastric cancer (32). Ding et al. also concluded that *HP*-negative gastric cancer occurs due to disruption of the normal microbiota, with elevated levels of some bacteria with pro-cancer activity and decreased levels of cancer-suppressing bacteria (33). In other words, it is not a certain kind of bacteria that causes gastric cancer alone. Instead, the microbiota in the stomach undergoes dysbiosis, and the various dysbiotic microbiota work together to participate in the development of gastric cancer.

## Gastric carcinogenesis

Numerous studies have found a connection between the development of gastric cancer and exogenous infections, such as *HP* and EBV, as well as endogenous dysbiosis. A shared mechanism among these factors is the induction of chronic inflammation, which heightens the risk of cancer (34). Chronic inflammation is vital in all three stages of gastric cancer development, from AG to IM and finally to gastric cancer. Chronic inflammation leads to the accumulation of mutations and the destabilization of the host genome by accelerating DNA replication rates and diminishing DNA damage repair capabilities. The result is pro-oncogenes’ activation and tumor suppressor genes’ (TSG) inactivation, ultimately leading to gastric cancer development (35).

### Exogenous pathogens

#### *HP* and its potential mechanism in gastric carcinogenesis

*HP*, a representative exogenous bacteria in the stomach’s microecology, can be transmitted through saliva, vomit, and feces. The carcinogenic mechanism of *HP* is closely linked to its virulence factors, which include cytotoxin-associated gene A (CagA), vesicular cytotoxin A (VacA), neutrophil-activating protein (NAP) and outer membrane proteins (OMPs) (3) **(** **Table 2****)**. These factors damage DNA in gastric epithelial cells and cause the destabilization of the host genome.

**Table 2:** Virulence factors closely associated with the carcinogenic mechanism of *HP*.

	Virulence factors	Mechanisms	Functions
**Pathogenic factors**	CagA (36, 37)	• Transfection into cytoplasm by T4SS • Phosphorylation of tyrosine	• Disrupts intercellular junctions • Facilitates inflammation • Causes cell proliferation • Promotes EMT
	VacA (38, 39)	• Selective anion channel properties • Targets different organelles	• Promotes *HP* colonization • Induces apoptosis and necrosis • Causes cell vacuolization • Enhances autophagy
	NAP (40–43)	• Overactivation of ERK and p38-MAPK pathways • DNA-binding protein • Structurally similar to bacterial ferritin	• Activates neutrophils, monocytes, and mast cells • Produces ROS • Promotes neutrophils’ adhesion to gastric epithelial cells • Promote the release of IL-8, IL-6, TNF-α, MIP-1α, MIP-1β, and β-hexosaminidase • Damages the gastric mucosa • Accelerates the process of chronic gastritis • Protects *HP* DNA from oxidative damage • Promotes the growth and colonization of *HP*
	HtrA (44, 45)	• Serine proteases • Chaperones	• Cleaves E-cadherin • Disrupts intercellular junctions • Disrupt the epithelial barrier • Enhances CagA translocation • Removes the wrong protein
	gGT (46–48)	• Promotes biochemical reactions • Glutamine hydrolysis	• Damages the gastric epithelium • Causes Glutathione consumption • Produces ROS • Causes cell cycle arrest • Induces apoptosis and necrosis • Induces immune tolerance • Up-regulates IL-8, caspase-3, caspase-9, and COX-2 • Enhances VacA-Dependent Vacuolation
**Adhesion factors**	BabA (49, 50)	Binds to Leb receptors on gastric epithelial cells	• Facilitates *HP* adhesion and colonization • Enhances CagA translocation • Activates inflammation
	SabA (51)	Binds to the sialyl-Lex antigens	• Facilitates *HP* adhesion and colonization • Promotes neutrophil activation and infiltration • Induces oxidative damage • Causes inflammation
	OipA (52–54)	Unknown	• Facilitates *HP* adhesion • Inhibits DC maturation • Induces apoptosis • Causes neutrophilic infiltration • Mediates CagA translocation • Increases IL-8 secretion • Decreases IL-10 expression
	HopQ (55–57)	Binds to the CEACAM receptors	• Facilitates *HP* adhesion • Enhances CagA translocation • Promotes the release of IL-8 and MIP-1α • Inhibits immune cell activities • Protects tumor cells
	HopZ (58, 59)	Unknown	• Facilitates HP adhesion • Causes infection • Inhibits gastric acid secretion

CagA, Cytotoxin-Associated Gene A; VacA, Vesicular Cytotoxin A; NAP, Neutrophil-Activating Protein; HtrA, High Temperature Requirement A; gGT, γ-Glutamyl Transpeptidase; BabA, Blood Group Antigen-Binding Adhesin; SabA, Sialic Acid-Binding Adhesin; OipA, Outer Inflammatory Protein; Hop, Helicobacter pylori Outer Membrane Protein; T4SS, Type 4 Secretion System; EMT, Epithelial-Mesenchymal Transition; *HP*, *Helicobacter pylori*; ROS, Reactive Oxygen Species; DC, Dendritic cell.

CagA, an oncoprotein, interacts with various signaling pathways upon entering the cytoplasm of gastric epithelial cells, disrupts intercellular junctions, and activates pro-inflammatory and oncogenic signaling pathways. This process leads to sustained inflammatory responses and uncontrollable cell proliferation. CagA can be transfected into the cytoplasm of gastric epithelial cells *via* the type 4 secretion system (T4SS) encoded by the Cag pathogenicity island (CagPAI) (2). CagA can regulate signal transduction through both phosphorylated and non-phosphorylated pathways. Phosphorylated CagA can directly bind or recruit the phosphatase SHP2 to activate the MAPK/ERK pathway. SHP2 is a key protein in CagA pathogenesis and can regulate ERK signaling in a non-Ras-dependent manner (36). Non-phosphorylated CagA, when combined with E-cadherin, decreases E-cadherin expression and promotes epithelial-mesenchymal transition (EMT), accelerating gastric cancer progression (37).

VacA possesses selective anion channel properties that facilitate the release of bicarbonate and organic anions into the cytoplasm, contributing to *HP* colonization in gastric epithelial cells and leading to apoptosis (36). Moreover, *HP* infection can induce gastric carcinogenesis through hypermethylation silencing of multiple TSGs, such as miR-124a-1, miR-124a-2, and miR-124a-3 (60). Abnormal alterations in the methylation levels of the CpG island of TSG promoters following *HP* infection affect the transcription of downstream genes, causing irreversible TSG inactivation (61).

NAP is the main pro-inflammatory factor in *HP* infection. The protein was initially named for its ability to activate neutrophils to produce Reactive Oxygen Species (ROS) and promote neutrophils’ adhesion to gastric epithelial cells (62). In addition to neutrophils, NAP activates monocytes and mast cells to release a variety of pro-inflammatory chemokines such as IL-8, IL-6, TNF-α, MIP-1α, MIP-1β, and β-hexosaminidase (63). The release of these factors causes damage to the gastric mucosa and accelerates the process of chronic gastritis. In addition, NAP has been found to play an essential role in bacterial protection. NAP is a DNA-binding protein belonging to the Dps family and has structural similarity to bacterial ferritin. Therefore, NAP can isolate free iron and bind DNA, which can protect *HP* DNA from oxidative damage and promote the growth and colonization of *HP* (40).

The most studied adhesins among *HP* outer membrane proteins are blood group antigen-binding adhesin (BabA) and sialic acid-binding adhesion (SabA). They can bind to receptors on the surface of gastric epithelial cells, and this binding contributes to *HP* adhesion and colonization, increases *HP* pathogenicity, and promotes persistent infection (64). BabA binds to Leb and enhances CagA translocation by promoting T4SS activity. CagA induces the production of massive pro-inflammatory factors, leading to IM and precancerous lesions (49). SabA binds to the sialyl-Lex antigen and promotes neutrophil activation and infiltration, inducing oxidative damage and causing a persistent inflammatory response (51). The expression of BabA and SabA is closely associated with the development and prognosis of various gastrointestinal diseases, such as chronic gastritis and gastric cancer (50, 65). Therefore, they may be potential targets for preventing and treating *HP*-related diseases.

**Figure 2:**
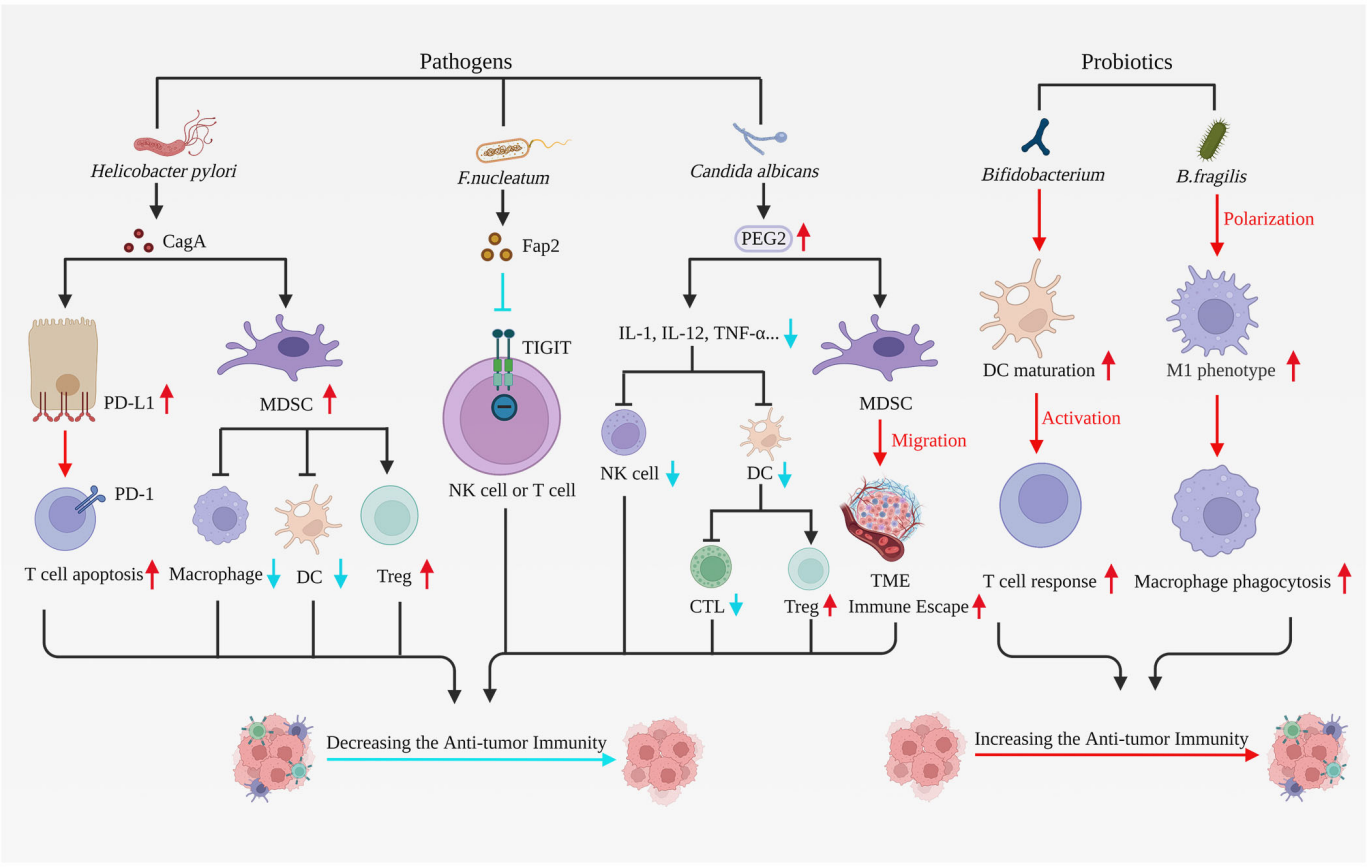
Pathogens and probiotics have effects on anti-tumor immunity. Pathogens correlated with gastric carcinogenesis comprise *Helicobacter pylori*, *F*. *nucleatum*, and *Candida albicans*, all of which can suppress immune cell function, stimulate tumor cell proliferation and hinder apoptosis, thereby fostering tumor advancement. *Helicobacter pylori* upregulate PD-L1 expression in gastric epithelial cells, consequently inducing T cell apoptosis. *Helicobacter pylori* additionally activate MDSCs, reducing DCs and macrophage function while enhancing Treg cell activity. The *F*. *nucleatum* virulence factor Fap2 obstructs tumor elimination by immune cells *via* binding and interacting with TIGIT on NK cells or lymphocytes. *Candida albicans* infection leads to heightened expression of the inflammatory factor PGE2. PGE2 diminishes cytokine levels such as IL-1, IL-12, and TNF-α, which restrain DC and NK cell activity and function, obstruct CTL activation, and promote Treg cell maturation, ultimately suppressing tumor immunity. Furthermore, PGE2 facilitates MDSC migration to TME, thus promoting tumor immune escape. Probiotics like *Bifidobacterium* and *B*. *fragilis* elicit robust immune responses, enhance anti-tumor immunity, and impede tumor progression. *Bifidobacterium* directly stimulates DC maturation and fosters immune response production by T cells. *B*. *fragilis* induces macrophage polarization toward the M1 phenotype and enhances macrophage phagocytic capacity. In summary, microbial pathogens associated with gastric carcinogenesis suppress anti-tumor immunity. However, probiotics can potentially enhance anti-tumor immunity, which has significant implications for the treatment and prognosis of gastric cancer. F. *nucleatum, Fusobacterium nucleatum; B. fragilis, Bacteroides fragilis;* MDSCs, myeloid-derived suppressor cells; Treg cells, regulatory T cells; TIGIT, T cell immunoreceptors with IG and ITIM domains; DC, Dendritic cell; TME, Tumor microenvironment. Created with BioRender.com. (accessed on 17 May 2023).

Additionally, *HP* infection may significantly diminish the effectiveness of immunotherapy for gastric cancer. Firstly, *HP* can decrease the activity of CD4+ T cells, dendritic cells (DCs), macrophages, and NKT cells, while also increasing the activity of myeloid-derived suppressor cells (MDSCs) and regulatory T cells (Treg cells) (66). Secondly, *HP* elevates the expression of PD-L1 in gastric epithelial cells and induces CD4+T cell apoptosis (67) **(** **Figure 2****)**. This finding implies that *HP* infection leads to non-specific suppression of T cells and reduced immune checkpoint inhibitors (ICIs) efficacy. Finally, Oster et al. discovered that *HP* infection could reduce the effectiveness of anti-CTLA-4 and anti-PD-L1 therapy, decrease the potency of cancer vaccines, and inhibit *in situ* tumor immunotherapy (66, 68).

#### EBV and its potential mechanism in gastric carcinogenesis

EBV, first isolated from Burkitt’s lymphoma cell line in 1964, was among the initial viruses identified to be linked to human malignancy. EBV infection also elevates the risk of developing gastric cancer by more than 18-fold, with EBV-associated gastric cancer constituting approximately 10% of all gastric cancers (69, 70).

Following EBV infection, viral genes such as EBERs, EBNA-1, and BARTs are expressed and can interact with host proteins to promote cell proliferation and inhibit apoptosis. The DNA damage and cell proliferation resulting from this process contribute to gastric cancer development. Additionally, EBV can disrupt the host’s epigenetic machinery and induce malignant transformation of the epigenome. EBV upregulates host DNA Methyltransferases (DNMTs) through the expression of Latent Membrane Protein 1 (LMP1) and Latent Membrane Protein 2A (LMP2A) viral oncoproteins (13, 71). Consequently, most EBV-associated gastric cancers exhibit CpG island hypermethylation at TSGs, such as PTEN, leading to the inactivation of TSGs and immune escape. Plasma EBV DNA load is a valid marker for assessing the efficacy and prognosis of EBV-associated gastric cancers (72). It has been discovered that EBV and *HP* may synergistically impact the development of gastric cancer. *HP*-positive patients have a higher EBV DNA load. EBV infection can enhance the oncogenic potential of CagA. Gastric cancers co-infected with EBV and *HP* display numerous methylated genes. EBV and *HP* can synergistically promote chronic inflammation and increase tissue damage during the early stages of the gastric carcinogenesis process (2, 73). Immunotherapy is typically employed to treat patients with EBV-associated gastric cancer because EBV subtypes are positively associated with PD-L1 overexpression (35). Su et al. observed an increased cellular immune response and cytotoxicity in EBV-associated gastric cancer xenograft model mice through CRISPR/Cas9 system-mediated disruption of PD-1 on T cells (74).

Several studies have suggested that other viruses, such as JCV and HBV, may also play a crucial role in gastric cancer development (75). The oncogenic effect of JCV is primarily achieved through its encoded oncoprotein T-Ag. T-Ag can interact with tumor suppressor proteins like p53 and pRB and participate in gastric carcinogenesis through critical signaling pathways, such as Wnt/β-Catenin (76). HBV infection results in a 1.29-fold increased risk of gastric cancer, with the hepatitis B virus X protein (HBx) potentially playing a vital role in this process (77). Cui et al. discovered that HBV could induce gastric epithelial cell carcinogenesis through HBx (78). Moreover, chronic inflammation caused by HBV infection heightens the risk of gastric carcinogenesis (79).

### Endogenous dysbiosis

#### *Lactobacillus* and its potential mechanism in gastric carcinogenesis

Although *Lactobacillus* is widely regarded as a probiotic in the intestine, it is often abundantly enriched in gastric cancer, particularly in advanced gastric cancer (32, 80). Several factors contribute to *Lactobacillus* causing gastric cancer. First, lactate, produced by *Lactobacillus*, plays a massive role in the Warburg effect of tumors (81). Second, *Lactobacillus* can upregulate pro-inflammatory genes, such as Ptger4 and Tgf-β, and oncogenic genes, such as Nos2, Tnf-α, Cxcl1 (Kc), and Ccl2 (Mcp-1) to induce gastric carcinogenesis (30). Third, *Lactobacillus* can reduce nitrate to nitrites and eventually form nitrosamines (82). Furthermore, *Lactobacillus* is a potent ROS inducer, leading to DNA damage (83). Lastly, *Lactobacillus* can convert human fibroblasts into multipotent cells, directly promoting gastric cancer development (84). These factors may jointly contribute to gastric cancer development, but the exact mechanism requires further investigation.

#### *F. nucleatum* and its potential mechanism in gastric carcinogenesis

*Fusobacterium nucleatum* (*F. nucleatum*) is most widely known for its carcinogenic effect on colorectal cancer. However, in recent years, *F. nucleatum* has also been recognized as a significant cause of gastric cancer development. *F. nucleatum* is more abundant in gastric cancer than normal tissues, and *F. nucleatum* positivity is associated with a worse prognosis and shorter overall survival in patients with diffuse-type gastric cancer (85, 86). *F. nucleatum* binds to E-cadherin in gastric epithelial cells *via* the characteristic virulence factor FadA, increasing endothelial permeability, promoting self-colonization, propagation, and immune escape (87), and activating the Wnt/β-catenin signaling pathway to enhance tumor cell proliferation (88). Familial adenomatous polyposis 2 (Fap2) is another virulence factor of *F. nucleatum*. Fap2, through binding and interaction with T cell immunoreceptors with IG and ITIM domains (TIGIT), can inhibit the killing of NK cells and lymphocytes to tumors, thereby protecting tumors and promoting the formation of an inflammatory microenvironment (89, 90). *F. nucleatum* also produces an induced inflammatory response through NF-κB-mediated cytokines such as IL-6, IL-1β, IL-17, IFN-γ, and TNF-α (87, 91).

#### *Candida albicans* and its potential mechanism in gastric carcinogenesis

Zhong et al. conducted ITS rDNA gene analysis on cancerous and non-cancerous tissues from 45 gastric cancer patients and discovered an imbalance of fungal communities in gastric cancer. After statistical analysis, they determined that *Candida albicans* were abundantly enriched in gastric cancer. Ultimately, it was demonstrated that *Candida albicans* could serve as a fungal biomarker for diagnosing gastric cancer. *Candida albicans* may promote gastric cancer development by reducing the diversity and abundance of fungi in the stomach, such as *Candida glabrata*, *Aspergillus montevidensis*, *Saitozyma podzolica*, and *Penicillium arenicola* (92).

*Candida albicans* may possess multiple carcinogenic mechanisms, including damage to mucosal epithelial cells, induction of chronic inflammation, and production of carcinogens leading to cancer.

Upon invasion of the mucosal epithelium, *Candida albicans* cause apoptosis and necrosis, which disrupts the immune barrier of the epithelium and leads to structural changes in the epithelium (93). Long-term colonization and infestation of *Candida albicans* in the mucosal epithelium can cause chronic inflammation. During this process, the expression of the inflammatory factor prostaglandin E2 (PGE2) increases. PGE2 downregulates macrophage cytokines, inhibits DC and NK cell activity and function, blocks cytotoxic T cell activation, and promotes maturation and suppressive activity of Treg cells, thereby suppressing tumor immunity. PGE2 aids in the migration of MDSCs to the tumor microenvironment, proliferation of malignant cells, and inhibition of apoptosis, directly promoting tumor progression. PGE2 can also promote tumorigenesis through ROS production, activation of oncogenic transcription factors, and promotion of angiogenesis (94).

*Candida albicans*-produced nitrosamines and acetaldehyde can contribute to cancer development to some extent. Acetaldehyde impacts DNA replication and can cause point mutations of genes and chromosomal abnormalities. Meanwhile, acetaldehyde interferes with enzymes involved in cytosine methylation and DNA damage repair, resulting in proto-oncogene activation and cell cycle abnormalities, thereby promoting cancer development. Additionally, acetaldehyde can cause mitochondrial damage and induce apoptosis (93).

In conclusion, *Candida albicans* dysbiosis can increase the likelihood of cancer occurrence and promote cancer progression.

## Microbiota from other habitats and gastric cancer

### Oral microbiota and gastric cancer

The oral and gastric environments are linked through swallowed saliva and ingested food. The oral microbiota is a major source of gastric microbiota (95). Many studies have found that dysbiosis of the oral microbiota is associated with gastric carcinogenesis. Oral bacteria such as *Parvimonas*, *Eikenella* and *Prevotella-2* were significantly enriched in gastric cancer compared to other precancerous stages (18). The abundance of oral bacteria such as *Peptostreptococcus*, *Streptococcus*, and *Fusobacterium* was higher in cancerous tissues than in paracancerous tissues (23). Network analysis revealed that oral microorganisms such as *Peptostreptococcus stomatitis*, *Slackia exigua*, *Parvimonas micra*, *Streptococcus anginosus*, and *Dialister pneumosintes* might play critical roles in the development of gastric cancer (25). Under normal conditions, most of the oral microbiota entering the stomach is either destroyed by gastric acid or protected by the mucus-bicarbonate barrier from invading the gastric epithelium. However, certain disease states, such as gastric ulcers and gastric cancer, can damage the gastric mucosa and neutralize gastric acid, allowing oral bacteria to colonize the gastric mucosa from gastric juices (17). Recently, some studies have also been on the interaction between oral microbiota and *HP*. On the one hand, *HP* infection can disrupt the homeostasis of the oral microbiota (96). On the other hand, ectopic colonization on the gastric mucosa by the oral microbiota may, in turn, be necessary for *HP*-induced ecological dysbiosis (95).

### Gut microbiota and gastric cancer

Since the pH in the intestine is more conducive to microbial colonization, intestinal bacteria are nearly 10^7^ times more abundant than those in the stomach (16, 97). Approximately 99% of the 10^14^ microorganisms that comprise the human microbiome reside in the intestine (98). Numerous studies have demonstrated that intestinal flora can directly and indirectly affect the development, treatment, and prognosis of gastric cancer.

In a study conducted in Shanxi province, researchers investigated changes in the gut microbiota of 116 patients with gastric cancer and 88 healthy controls. They observed an increase in the abundance of intestinal flora, a decrease in butyrate-producing bacteria, and a significant enrichment of *Lactobacillus*, *Escherichia*, and *Klebsiella* in patients with gastric cancer (99). According to Sarhadi et al., *Enterobacteriaceae* were *abundant* in stool samples from patients with all types of gastric cancer (100). *Enterobacteriaceae* may serve as a potential biomarker in the diagnosis of gastric cancer.

Certain metabolites of intestinal bacteria, such as acetic acid and butyrate, also influence the development of gastric cancer. Acetic acid and butyrate are the major short-chain fatty acids, and intestinal bacteria such as *Eubacterium*, *Clostridium*, *Ruminococcus*, and *Coprococcus* can produce butyrate (35). Increasingly, research has found that butyrate plays a crucial role in inhibiting gastric cancer development. First, butyrate inhibits the Warburg effect of gastric cancer by binding pyruvate kinase M2 (PKM2), increasing the content of glucose intermediates in mitochondria, preventing the conversion of tricarboxylic acid cycle intermediates to ATP, and ultimately depriving tumor growth of sufficient energy, thus inhibiting gastric cancer development. Second, butyrate can directly interfere with the mitochondria of gastric cancer cells, upregulate oxidative stress, and significantly increase the level of ROS. Moreover, butyrate can promote caspase 9 production and inhibit BCL-2 synthesis, leading to the apoptosis of gastric cancer cells (101). Lastly, a recent study discovered that butyrate could inhibit the growth, migration, and invasion of gastric cancer cells and aerobic glycolysis by blocking the Wnt/β-catenin/c-Myc signaling pathway (102).

## Microbiota modification and immunotherapy for gastric cancer

Alterations in the microbiota may directly impact gastric cancer immunotherapy (103, 104). Matson et al. discovered that higher proportions of *Bifidobacterium longum*, *Collinsella aerofaciens*, and *Enterococcus faecium* led to more effective anti-PD-L1 therapy (105). A meta-analysis revealed that *Bacteroides caccae* was enriched in all types of immune checkpoint inhibitor therapies. *Faecalibacterium prausnitzii*, *Bacteroides thetaiotamicron*, and *Holdemania filiformis* were enriched in responders to anti-CTLA-4 and anti-PD-1 ICIs treatments (106). Based on these findings, researchers have proposed microbiota modification as a means to enhance the efficacy of immunotherapy for gastric cancer. Current approaches to microbiota modification include the use of probiotics and FMT.

### Probiotics

Probiotics, such as *Bifidobacterium*, *Lactobacillus*, and *Saccharomyces*, are active microbes that benefit the host’s health (107, 108). There is growing evidence that probiotics may inhibit the growth of gastric cancer to some extent. Probiotics can significantly reduce inflammatory responses, enhance the immune system, promote tumor cell apoptosis, restore gut microbiota homeostasis, and inhibit cancer signaling pathways (35, 109, 110). *Lactobacillus* can inhibit the development of gastric cancer by reducing the expression of NF-κB and the phosphorylation of the PI3K/Akt/mTOR signaling pathway (111). *Bifidobacterium* can directly induce DC maturation and cytokine (IFNγ, TNFα, IL-10, IL-17) production, thus promoting anti-tumor immunity and anti-PD-L1 efficacy and almost inhibiting tumor progression after combination therapy with PD-L1 monoclonal antibody (mAb) (104). *Bacteroides thetaotaomicron* and *Bacteroides fragilis* (*B. fragilis*) are also potential probiotics. Vétizou et al. found that the antitumor and immunotherapeutic effects of CTLA-4 blockade were associated with *Bacteroides thetaotaomicron* and *B. fragilis*. Oral administration of *Bacteroides thetaotaomicron* or *B. fragilis* to germ-free mice enhances the antitumor effects of CTLA-4 blockers (112). *B. fragilis* also increase macrophage phagocytosis, prompting them to polarize towards an M1 phenotype (113).

Probiotics have become a prominent research topic due to their beneficial effects on gastric cancer. Immunotherapeutic probiotics could be developed to improve the efficacy of immunotherapy (114). However, contrary to the aforementioned findings, it is relatively easy to identify inconsistencies. The role of *Lactobacillus* in gastric cancer remains unclear. In addition to *Lactobacillus*, there are inconsistent findings on the effects of other probiotics on gastric cancer. Based on human microbiome studies and animal models, researchers have cautioned against the direct use of Lactobacillus in treating patients with gastric cancer (81).

### FMT

FMT is transplanting fecal material from a healthy donor into a recipient’s intestine, ultimately altering the recipient’s intestinal flora to match the donor’s microbial profile (115). Several methods of fecal microbiota transplantation exist, including oral, nasal, and rectal administration. Rectal administration is preferred, as nasal administration may cause pulmonary or gastrointestinal complications, and although oral administration is convenient, careful avoidance of first-pass effects is necessary (116, 117). Given FMT’s numerous benefits, there is growing interest in its potential for enhancing cancer immunotherapy.

Routy et al. discovered that the aberrant composition of intestinal flora results in primary resistance to immune checkpoint inhibitors (ICIs) in patients with late-stage cancer and that antibiotics interfere with the therapeutic efficacy of ICIs in patients. Their findings revealed that the anti-tumor effects of PD-1 inhibitors are more effective in germ-free mice or those not treated with antibiotics after FMT from cancer patients who respond to ICIs. However, this was not the case in the FMT of patients who did not respond to ICIs. They also analyzed the metagenomics of fecal material from patients and found a correlation between the efficacy of ICIs and the abundance of *Akkermansia muciniphila* (*A. muciniphila*). After FMT, patients who did not respond were given *A. muciniphila* can restore the efficacy of PD-1 inhibitors (103). Fecal material from immunotherapy responders was transplanted into germ-free mice, resulting in slower tumor growth and increased immunotherapy efficacy (105). In summary, FMT can enhance patient responses to immunotherapy.

Since FMT has a more substantial impact on intestinal flora than gastric flora, most current research focuses on the effect of FMT on intestinal diseases. However, researchers should also consider FMT as a treatment for gastric cancer and precancerous lesions in the stomach. As a safe and effective therapy, FMT may treat gastric cancer in the future by interfering with the composition of the intestinal flora. However, we need further studies to evaluate FMT’s safety and reduce the risk of side effects.

## Other interventions and therapeutic strategies for gastric cancer treatment

In addition to the FMT and probiotics mentioned above, many potential microbial interventions have been used to treat gastric cancer, including diet, prebiotic, and bacteriophage-based strategies, all pointing to the promising future of microbial therapies.

### Bacteriophage-based strategies

Bacteriophages are viruses that selectively infect bacteria (118) and are more than 100 times more abundant in the gut than in bacteria and human cells (119). Given that most bacteriophages are particular to specific pathogenic bacteria but do not disrupt the homeostasis of normal flora (120), many studies have constructed bacteriophage-based biotic–abiotic hybrid nanosystems to accurately remove tumor microorganisms (121).

In order to precisely regulate intestinal flora to treat colorectal cancer, Zheng et al. loaded irinotecan inside dextran nanoparticles (122). And through bioorthogonal reaction, it was covalently linked with azide-modified phages that could specifically lyse *F. nucleatum*, which could not only effectively inhibit the proliferation of *F. nucleatum*, but also reduce the toxic side effects caused by the non-targeted release of conventional chemotherapeutic drugs. Moreover, phages can also remodel TME. *F. nucleatum* selectively amplifies MDSCs, thereby blocking the body’s anti-tumor immune response. Through phage display technology, Dong et al. screened M13 phage that can specifically bind *F. nucleatum* (123). They further assembled silver nanoparticles on the surface capsid protein of the M13 phage (M13@Ag). Subsequently, it was also demonstrated that M13@Ag could specifically clear *F. nucleatum* and inhibit the proliferation of MDSCs, reversing the immunosuppressive TME. In addition, the M13 phage utilizes its coat protein to activate antigen-presenting cells, which further activates the host immune response and inhibits CRC. Animal experiments demonstrated that M13@Ag combined with immune checkpoint inhibitors or chemotherapeutic agents significantly prolonged the survival of CRC mice. A recent study on phage strategies targeting *HP* may open new avenues for treating *HP*-positive gastric cancer (124).

### Diet and prebiotic strategies

Diet has an important influence on the composition of the gut microbiota. Different dietary habits can lead to differences in the composition of the gut microbiota (125). Compared to omnivores, the intestinal flora of strict vegetarians was characterized by a higher abundance of Bacteroidetes and a lower abundance of Firmicutes (126). However, the opposite alteration of the intestine flora was observed in people on chronic high-fat diets (127). Therefore, the composition of the intestinal flora can be adjusted by dietary interventions, thereby creating a more favourable microecological environment for the host. Dietary interventions such as fasting and calorie restriction have been used in clinical trials for various cancers, and the results have shown that dietary interventions can alter the systemic metabolism of cancer patients, improve anti-tumor immunity (128), and inhibit tumor growth. For example, in an ongoing clinical trial (NCT01642953), researchers restrict patients’ diets after gastric cancer surgery to assess whether fasting promotes recovery and reduces mortality and adverse events (129). Dietary interventions can also inhibit inflammation, improve anti-cancer immune surveillance (130) and prevent chemotherapy-induced toxic side effects (131).

WHO defines the concept of prebiotics as “an inactive food ingredient that promotes the health of the organism by modulating the composition of the microbiota (107).” Common prebiotics are fructooligosaccharides (FOS), galactooligosaccharides (GOS) and inulin (132). Prebiotics can help the body resist pathogenic infections, maintain stable gastrointestinal function, promote the production of short-chain fatty acids by beneficial bacteria, regulate immunity and energy metabolism, and increase the absorption of micronutrients, thus improving the efficacy of cancer treatment (132–134). To demonstrate the role of prebiotics in cancer treatment, many preclinical studies and clinical trials are underway or have been completed. One clinical trial in the United States (NCT04682665) is investigating eicosapentaenoic acid’s role in treating liver metastases from colorectal cancer (135). Furthermore, It has been shown that a high intake of Ganoderma spp (136). Polysaccharides and raffinose (137) potentially reduce the risk of gastric cancer, but more extensive independent studies and clinical trials are needed to confirm this.

## Conclusions and perspective

The microbiota has dual effects on gastric cancer. On the one hand, pathogenic bacterial infections and dysbiosis can promote the development of gastric cancer and is detrimental to the treatment and prognosis of gastric cancer. On the other hand, microbial therapies that optimize the microbiota through dietary changes, the use of bacteriophages and probiotics, and even transplantation of the fecal microbiota hold promise as potential treatments for gastric cancer. Furthermore, specific microorganisms and metabolites in gastric and intestinal flora could potentially serve as markers for diagnosing and monitoring the prognosis of gastric cancer, thereby improving early detection and treatment. Studying the microbiota helps us further understand the mechanisms of gastric cancer development and may provide new ideas for targeting microbiota interventions to treat cancer. However, there are still some problems in the current research on the microbiota of gastric cancer. Firstly, the mechanism by which some microbes promote gastric cancer has not been conclusively demonstrated and requires further investigation. Second, due to the differences in sequencing technology, analysis method or study population, the results obtained by many studies are quite different, and the interference of these factors should be excluded as far as possible. Third, there is currently a lack of more definitive biomarkers for gastric cancer other than prevention or eradication of *HP* infection, which hinders the early diagnosis of gastric cancer. In the future, more convenient and efficient biomarkers should be explored in larger sample sizes. In conclusion, further exploration is needed to modify the microbiota and ultimately improve the prognosis and effectiveness of gastric cancer treatment.

## Author contributions

JX conceived and designed the review. YW and NW wrote the draft of the paper. WH and MH collected the information. YW and MB drew the picture. YD, JD, TS, and JX participated in the manuscript revision. All authors read and approved the final manuscript and submission of this manuscript. All authors contributed to the article and approved the submitted version.
